# Ethical Guidance for Hard Decisions: A Critical Review of Early International COVID-19 ICU Triage Guidelines

**DOI:** 10.1007/s10728-021-00442-0

**Published:** 2021-10-26

**Authors:** Yves Saint James Aquino, Wendy A. Rogers, Jackie Leach Scully, Farah Magrabi, Stacy M. Carter

**Affiliations:** 1 Australian Centre for Health Engagement, Evidence and Values, School of Health and Society, University of Wollongong, Northfields Ave, Wollongong, NSW 2522 Australia; 2 Department of Philosophy and Department of Clinical Medicine, Macquarie University, Macquarie Park, NSW 2109 Australia; 3 Disability Innovation Institute, University of New South Wales, Sydney, NSW 2052 Australia; 4 Australian Institute of Health Innovation, Macquarie University, Macquarie Park, NSW 2109 Australia

**Keywords:** COVID-19, Ethics, Resource allocation, Triage, Intensive care

## Abstract

This article provides a critical comparative analysis of the substantive and procedural values and ethical concepts articulated in guidelines for allocating scarce resources in the COVID-19 pandemic. We identified 21 local and national guidelines written in English, Spanish, German and French; applicable to specific and identifiable jurisdictions; and providing guidance to clinicians for decision making when allocating critical care resources during the COVID-19 pandemic. US guidelines were not included, as these had recently been reviewed elsewhere. Information was extracted from each guideline on: 1) the development process; 2) the presence and nature of ethical, medical and social criteria for allocating critical care resources; and 3) the membership of and decision-making procedure of any triage committees. Results of our analysis show the majority appealed primarily to consequentialist reasoning in making allocation decisions, tempered by a largely pluralistic approach to other substantive and procedural values and ethical concepts. Medical and social criteria included medical need, co-morbidities, prognosis, age, disability and other factors, with a focus on seemingly objective medical criteria. There was little or no guidance on how to reconcile competing criteria, and little attention to internal contradictions within individual guidelines. Our analysis reveals the challenges in developing sound ethical guidance for allocating scarce medical resources, highlighting problems in operationalising ethical concepts and principles, divergence between guidelines, unresolved contradictions within the same guideline, and use of naïve objectivism in employing widely used medical criteria for allocating ICU resources.

## Introduction

The pandemic spread of SARS-CoV2 from late 2019 led to many countries experiencing high demand for acute and critical care in 2020 [1]. Surges in hospital admissions during both first and subsequent waves were predicted to overwhelm, and in some cases (e.g. China [2], Italy [3], the US [4], and France [5]) did overwhelm, the capacity of intensive or critical care units (ICUs) in many countries. Since the initial outbreak, subsequent waves of infection have triggered reinstatement of restrictions and repeat surges in hospital admissions [5, 6]. Affected countries report varying impacts such as cancellation of non-COVID-related healthcare services [7], increasing mortality[2], repurposing of hospital units and staff, and extensive use of public health restrictions to reduce transmission and thus ICU demand [8].

The volume of COVID-19 cases requiring hospital admission raises ethical issues with regard to rationing health care resources. Periods of disaster and public health crisis tend to upend traditional priorities, shifting towards the utilitarian goal of saving the largest number of lives. In non-emergency conditions in high-income countries with accessible healthcare systems, decisions about ICU care are usually made by ICU specialists in consultation with the patient and/or relevant decision makers including family and friends. These decisions take account of the patient’s condition and prognosis as well as their preferences about the nature and extent of care they would like, with the proviso that patients do not usually have a right to demand non-beneficial ICU care. Decisions take place within the well-accepted ethical parameters of respecting patient autonomy and balancing the potential benefits of treatment against possible harms and futility. Issues of distributive justice in access to ICU beds rarely arise at the level of individual patient care in high-income countries, as there is normally sufficient capacity to meet need.

In the COVID pandemic, the numbers of patients who might benefit from ICU care has, in many high-income countries, outstripped availability of beds, forcing clinicians to make decisions about who may access intensive care among all of those that might benefit. In response to the intensity and urgency of the situation caused by the pandemic, there were calls for, and subsequent proliferation of, protocols, tools and guidance for allocating scarce ICU resources during the COVID-19 pandemic [9, 10]. While some of the tools explicitly drew upon pre-existing resources for decision making in situations of scarcity (such as crisis standards of care), a large number of COVID-19-specific protocols and decision aids were released by national, local and professional bodies to assist with this decision making. These tools are based on varying combinations of medical, ethical and social criteria, with a general focus on maximising the number of lives saved [11, 12].

The proliferation of COVID-19 triage guidelines has triggered secondary ethical analyses [13–15]. A US study of the ventilator triage policies of 29 hospitals found substantial heterogeneity, with many policies lacking guidance on fair implementation [13]. A second study systematically reviewed 31 US state crisis standards of care documents against five key ethical elements recommended by the Institute of Medicine [14]. That study found considerable heterogeneity among the plans and concluded that many US states have inadequate guidance to inform providers and policy-makers about effective strategies for allocating scarce resources during a public health crisis.

Several ethical analyses of international guidelines for allocating ICU resources during the COVID-19 pandemic have since been published [15, 16]. Jöbges et al., for example, found that justice and maximizing benefit were the two normative foundations of the 11 guidelines they reviewed, and they concluded by recommending largely procedural ethical guidance for ensuring that benefits are maximized under conditions of equity and equality. Jöbges et al. focus on the content of guidelines available in English in March–April 2020, and provide little detail on their search strategy.

Given existing scholarship in the area, our aim was to undertake a comprehensive search for and critical comparative ethical analysis of the resulting guidelines, including details about guideline development. We first identify the extent to which guidelines are explicitly based on accepted ethical concepts. Second, we evaluate how the ethical concepts are operationalised to inform decisions in practice, identifying areas of convergence and disagreement. Finally, we identify challenges in using ethical concepts within guidance documents; discuss the problematic notion of objectivity underpinning reliance on illness severity scores; and document and consider the practical and theoretical implications of our study. Our paper is distinctive in that it provides a level of analysis not offered in the existing literature. Specifically, we identify and discuss the challenges of operationalizing abstract concepts and dealing with inconsistencies in the guidelines; the problematic notion of objectivity used in the guidelines; and the practical and theoretical implications of guidelines that give divergent advice.

## Methods

We aimed to construct a complete set of local and national guidelines for allocating scarce resources during the COVID-19 pandemic, within specified criteria. Our specific focus was on guidance that is actionable, i.e. intended for use by clinicians to make decisions about the care of specific patients.

**Fig. 1:**
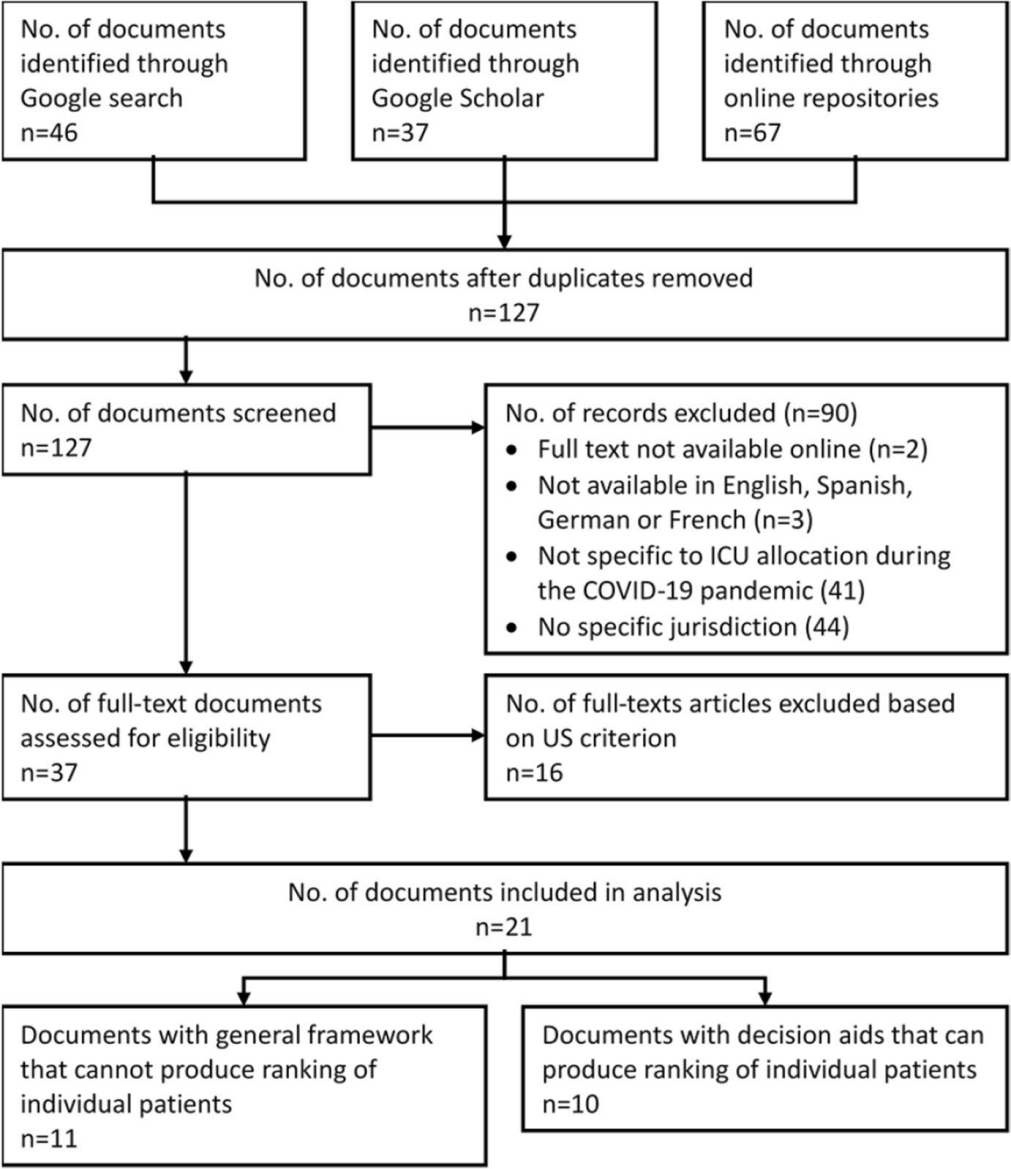
PRISMA flow diagram showing the number of documents included and excluded based on several criteria

Three search strategies were used to locate relevant guidelines. The first was a Google search using the terms “allocation” “guideline” “COVID” and “ICU” (with no conjunctions, defaulting to an “and” function). A separate query used the phrase “crisis standards of care”, results of which were added to the first Google search. Second, Google Scholar was searched using the same terms as the Google search. Third, online repositories of COVID-19 resources were searched for guidelines with titles that included the phrase COVID-19 allocation guideline or framework [17–23]. Repositories such as those compiled by the World Health Organization, European Commission and the Hastings Center contain current ethical, medical and legal guidance for the management of COVID-19. YSJA performed the searches in May 2020, with a final update on 18 June 2020 (see Fig. 1 for summary of search strategy). We abandoned our initial attempt to use common literature databases such as PubMed as the relevant guidelines are either living documents or not published in peer-reviewed journals.

These searches identified a total of 150 documents, with 127 guidelines remaining after removal of duplicates. These were screened for eligibility based on title and abstract (or introduction if abstract was not available). A total of 37 guidelines met the eligibility criteria of: online availability; in English or other languages available to researchers (i.e. Spanish, German and French); applicable to a specific and identifiable jurisdiction; and providing guidance to clinicians for decision making when allocating critical care resources during the COVID-19 pandemic. The jurisdictional requirement was included because guidelines with specific jurisdictions are more likely to have traction in practice and be expressed as standards of care [24]. The requirement for a decision-making framework was included because the focus of this study is to identify which ethical concepts underpin decisions and how these are operationalized, rather than general guidance. A total of 90 guidelines were excluded as they did not meet any of the inclusion criteria. We then also excluded US guidelines (n = 16), given the two published studies discussed above [13, 14].

The final data set comprised 21 guidelines, 11 of which contained general advice for allocation of ICU resources and 10 of which contained advice and/or specific decision trees for guiding individual patient decisions. Two authors (YSJA and WR) ensured that these 21 guidelines met the inclusion criteria. We used versions of the guidelines that were current on 18 June 2020.

Data were extracted from the guidelines using an Excel spreadsheet. This was developed by all authors, and refined after pilot testing on 10 guidelines in May 2020. One author (YA) extracted the data. A second author (WR) screened 10% of the yields. Conflicting assessments were resolved by consultation between at least two authors.

Information was extracted from each guideline regarding: (1) the development process for the guideline including use of evidence and any stakeholder consultations; (2) the presence and nature of ethical, medical and social criteria for allocating critical care resources; and (3) the membership of and decision-making procedure for any triage committee recommended by the guidelines.

## Results

The final data set comprised 21 guidelines [25–45], 11 of which [25–28, 30, 32, 33, 42–45] contained general advice for allocation of ICU resources and 10 of which [29, 31, 34–41] contained specific decision trees for guiding decisions about individual patients (see Table 1). Authorship of the guidelines varied, with 11 authored by a medical specialty organisation, 8 by a government organisation (local and international) and 2 by a university or hospital. Guidelines are from 13 countries, namely Australia (n = 3), Germany (n = 2), Austria (n = 2), Italy (n = 2), Ireland (n = 1), Spain (n = 3), Canada (n = 2), United Kingdom, France, Switzerland, Belgium, South Africa and Philippines (all n = 1). Most guidelines are available in English (n = 14), with the remainder published in German (n = 3), French (n = 2) or Spanish (n = 2).

**Table 1:** List of included guidelines including the shortened name, author/s and document title

Shortened name	Author	Document title
ANZICSa	Australian and New Zealand Intensive Care Society (ANZICS)	ANZICS COVID-19 Guidelines, Version 1 [25]
ANZICSb	Australian and New Zealand Intensive Care Society (ANZICS)	Guiding Principles for Complex Decision Making during Pandemic COVID-19 [26]
BÄK Germany	German Medical Association (*Bundesärztekammer*)	German Medical Association’s Guidance for the Allocation of Medical Resources Using the Example of the SARS-CoV-2-Pandemic During Limited Capacity (*Orientierungshilfe der Bundesärztekammer zur Allokation medizinischer Ressourcen am Beispiel der* SARS-CoV-2-*Pandemie im Falle eines Kapazitätsmangels*) [27]
BK Austria	Austrian Bioethics Commission (*Bioethikkommission*)	Management of scarce resources in healthcare in the context of the COVID-19 pandemic, English translation [28]
CCSSA	Critical Care Society of Southern Africa	Allocation of Scarce Critical Care Resources during the COVID-19 Public Health Emergency in South Africa [29]
CNB Italy	Italian National Committee on Bioethics (*Comitato Nazionale per la Bioetica*)	COVID-19: Clinical Decision-Making in Conditions of Resource Shortage and the “Pandemic Emergency Triage” Criterion (COVID-19: *La Decissione Clinica in Condizioni di Carenza di Risorse e il Criterio del “Triage in Emergenza Pandemica”)* [30]
DIVI Germany	German Interdisciplinary Association for Intensive Care and Emergency Medicine (*Deutschen Interdisziplinären Vereinigung für Intensiv- und Notfallmedizin*)	Decisions on allocation of resources in the emergency and intensive care in the context of the COVID-19 pandemic (*Entscheidungen über die Zuteilung intensivmedizinische Ressourcen im Kontext der* COVID-19-*Pandemie*) [31]
DOH Ireland	Irish Department of Health	Ethical Framework for Decision-Making in a Pandemic [32]
MSCBS Spain	Spanish Ministry of Health (*Ministerio de Sanidad, Consumo y Bienestar Social*)	Ministry of Health Report on Ethical Issues in Pandemic Situations: SARS-CoV-2 [33]
MSSS Quebec	Quebec Ministry of Health and Social Services (*Ministère de la Santé et des Services Sociaux*)	Triage for Access to Intensive Care (Adult and Pediatric) and Allocation of Resources such as Ventilators During Extreme Situation of a Pandemic (*Triage pour l’accès aux soins intensifs (adultes et pédiatriques) et l’allocation des ressources tells que les respirateurs en situation extreme de pandémie*) [34]
NICE UK	National Institute for Health and Care Excellence (NICE), UK	COVID-19 Guideline: Critical Care in Adults [35]
ÖGARI	Austrian Society for Anaesthetsiology, Resuscitation and Intensive Care Medicine (*Österreichische Gesellschaft für Anaesthesiologie, Reanimation und Intensivmedizin*)	Allocation of intensive care resources during the COVID-19 pandemic (*Allokation intensivmedizinischer Ressourcen aus Anlass der Covid-19-Pandemie*) [36]
Ontario	Ministry of Health, Ontario, Canada	Clinical Triage Protocol for Major Surge in COVID Pandemic [37]
QLD Health	Queensland Health, Queensland, Australia	Queensland Ethical Framework to Guide Clinical Decision Making in the COVID-19 Pandemic [38]
RPMO France	Coordination of Epidemic and Biological Risk, France (*Coordination opérationnelle du risque épidémique et biologique*)	Ethical issues and strategies in access to resuscitation and critical care during the COVID-19 pandemic (*Recommandation professionnelle multi-disciplinaire opérationnelle (RPMO): Aspects éthiques et stratégiques de l’accès aux soins de réanimation et autres soins critiques (SC) en contexte de pandémie* COVID-19) [39]
SAMS	Swiss Academy of Medical Sciences (*Schweizerische Akademie der Medizinischen Wissenschaften/Académie Suisse des Sciences Médicales/Accademia Svizzera delle Scienze Mediche*)	COVID-19 Pandemic: Triage for Intensive-Care Treatment under Resource Scarcity [40]
SEMICYUC	Spanish Society of Intensive Care Medicine and Coronary Units (*La Sociedad Española de Medicina Intensiva, Crítica y Unidades Coronarias*)	Ethical Recommendations for Decision Making in the Exceptional Situation of Crisis due to Pandemic COVID-19 in Intensive Care Units (*Recomendaciones éticas para la Toma de Decisiones en la Situación Exceptional de Crisis por Pandemia* COVID-19 *en las Unidades de Cuidados Intensivos*) [41]
SIAARTI	Italian Society of Anaesthesia, Analgesia, Resuscitation and Intensive Care (*Società Italiana di Anestesia Analgesia Rianimazione e Terapia Intensiva*)	Clinical Recommendations for the Allocation of Intensive Care Treatments, in Exceptional, Resource-Limited Circumstances [42]
SIZ Belgium	Belgian Society of Intensive Care Medicine (SIZ Medicine)	Ethical Principles Concerning Proportionality of Critical Care during the 2020 COVID-19 Pandemic in Belgium: Advice by the Belgian Society of Intensive Care Medicine [43]
UB-OBD	Bioethics and Law Observatory, University of Barcelona, Spain (*Observatori de Bioètica i Dret*)	Recommendations for Ethical Decision-Making on the access of Patients to Intensive Care Units in Pandemic Situations (*Recomendaciones para la Toma de Decisiones Éticas sobre el Accesso de Pacientes a Unidades de Cuidados Especiales en Situaciones de Pandemia*) [44]
UP-PGH	University of the Philippines-Philippine General Hospital, Manila, Philippines	Ethics Guidelines on COVID-19 Crisis-Level Hospital Care [45]

### Development Process of the Guidelines

There are wide variations in the descriptions of each guideline’s development. Nine of the guidelines (42%) offer no information, apart from listing authors, on the process used to develop the document (see Table 2 below). Some guidelines briefly mention consultations with experts [25, 26, 30, 40] or other documents on which the guideline is based [29, 34]. The CCSSA [29] largely draws from the University of Pittsburgh Guidelines [46], while the MSSS Quebec [34] guideline is adapted from the Hamilton Health Science Critical Care Triage Policy [47]. Five documents [31, 33, 35, 37, 38] offer comprehensive details of development by describing methods of consultation and collaboration. The UK NICE guideline [35] refers to an online source describing the “interim process and methods for developing rapid guidelines on COVID-19” [48]. The QLD Health guideline [38] reports consultation with clinical and ethics experts, and also with organisations representing Aboriginal and Torres Strait Islander People, people with disability, older persons and culturally and linguistically diverse groups (CALD), among others.

**Table 2:** Description of guideline development process including level of detail, number of guidelines and specific guidelines that fall under the described level of detail

Level of detail	Number	Guidelines (GL)
Comprehensive details provided (information about consultation processes, groups consulted, authorship)	N = 5	DIVI Germany [31], MSCBS Spain [33], NICE[35], Ontario [37], QLD Health [38]
Some details provided (e.g. brief mention of consultation or listing of authors)	N = 7	ANZICSa [25], ANZICSb [26], CCSSA [29], CNB Italy [30], MSSS Quebec [34], RPMO France [39], SAMS [40]
No details provided, or only lists authorship with no description of the development of document	N = 9	BÄK [27], BK Austria [28], DOH Ireland [32], ÖGARI [36], SEMICYUC [41], SIAARTI [42], SIZ Belgium [43], UB-OBD [44], UP-PGH [45]

Four guidelines [25, 33, 35, 37] indicate an ongoing process of revision as the authors or the organisation receive new information or evidence. For example, the ANZICSa guideline [25] states that it is a “living document”^1^ and the MSCBS Spain guideline [33] clarifies that the recommendations “may be revised as necessary … in view of ongoing developments” (p. 2).

### Ethical Approaches and Concepts in Allocating Resources

Fifteen guidelines explicitly state their commitment to a broad ethical approach or framing in the allocation of limited ICU resources (see Table 3). Of these broad approaches, consequentialism is the commonest; thirteen guidelines [26, 29–34, 37, 38, 40–42, 44] expressly commit to maximising benefits for the greatest possible number of people. Four guidelines [26, 31, 33, 45] frame their guidance in terms of patient-centred care with a focus on caring for “every critically ill person” ([33], p.7). All guidelines, including those without an identifiable broad ethical approach, draw on widely-used ethical resources, such as Beauchamp and Childress’ principlism (e.g. SAMS[40]), deontology (e.g. CNB Italy [30]), and professional codes of ethics (e.g. BÄK [27]; see Table 3).

**Table 3:** Ethical approaches and concepts including example statements, count or number of guidelines mentioning the concept, and the guidelines mentioning the concept

Concepts	Examples from guidelines	Count	Guidelines
*A. Broad ethical approaches*			
Consequentialism	“A consequentialist approach that ensures the greatest benefit and least harm for the maximum number of people is justified ([26], p.5) “The overall purpose of a triage system is to minimise mortality and morbidity for a population overall.” ([37], p.2)	13	[26, 29–34, 37, 38, 40–42, 44]
Patient-centred care	“every critically ill person deserves to receive vital care that they need” ([33], p.7) “The comprehensive patient assessment should include discussions about goals of care, patient and family preferences, and the acceptability to the patient of critical care interventions if offered.” ([26], p.6)	4	[26, 31, 33, 45]
Other (e.g. principlism)	Principlism: “The four widely recognised principles of medical ethics (beneficence, non-maleficence, respect for autonomy and equity) are also crucial under conditions of resource scarcity.” ([40], p.2)	6	[27, 28, 30, 40–42]
*B. Substantive ethical concepts*			
Autonomy, self-determination, freedom	“Patients’ wishes in respect of ICU care need to be ascertained.” ([29], p.5) “advance healthcare directives or advance care planning should be carefully evaluated” ([42], p.5)	19	[25–31, 34–45]
Beneficence	“The duty to search for the best possible, even if not optimal, care for the well-being of the sick person” ([28], p.5) “All patients who meet usual medical indications for ICU beds and services will be assigned a priority score using a 1–8 scale (lower scores indicate higher likelihood of benefit from critical care)…” ([29], p.5)	14	[26, 27, 29–34, 37, 38, 40–42, 44]
Justice as equality	“everyone matters, everyone matters equally” ([38], p.8)	14	[25, 26, 28–30, 32, 36, 38–42, 44]
Justice as social justice/equity	“…some people are in need of special support to be able to effectively exercise their fundamental right to life and the access to associated medically indicated treatment” ([28], p.6) “general applicable criteria …non-discrimination for any reason beyond the patient’s clinical situation and their objective, evidence-based expectations of survival” ([33], p.3)	12	[25–30, 32–34, 37–39, 45]
Duty to provide care	“It is the task of physicians to preserve life, protect and restore health…” (“*Aufgabe der Ärztinnen und Ärzte ist es, das Leben zu erhalten, die Gesundheit zu schützen und wiederherzustelle*…”; [27], p.1)	11	[26, 27, 29, 30, 32, 33, 36, 39–41, 45]
Non-maleficence	“No harm, understood as the primary duty … not to carry out treatments that cause more harm than good for the individual patient” *(“Nichtschaden, verstanden als primäre Pflicht … keine Behandlungen durchzuführen, welche für die individuell betroffene Patientin mehr Schaden als Nutzen bringen*”; [36], p.5) “A foundational principle of public health ethics is the obligation to protect the public from serious harm” ([32], p.6)	10	[27, 28, 30, 32, 34, 36, 39–41, 45]
Solidarity	“As individuals we can express our solidarity with others by supporting those in need of help and making joint efforts to avert/reduce the threat. Protecting the public, and hence ourselves, will require society-wide collaboration e.g. practicing good respiratory etiquette, hand hygiene or staying at home when ill” ([32], p.3) “Solidarity calls for a collaborative approach to pandemics that set aside conventional ideas of self-interest or territoriality at every level of society” ([32], p.6)	9	[25, 26, 28, 30, 32, 33, 38, 41, 45]
Rights	“health is referred to as a ‘fundamental right of the individual’” ([30], p.4) “Individuals have a right to privacy and confidentiality with respect to their health information” ([32], p.7)	8	[28, 30, 32, 33, 38, 39, 44, 45]
Stewardship	“prudent stewardship … requires prudent balancing of current patients’ needs with stewardship of resources” ([45], p.5) “Stewardship – that leaders arrive at decisions based upon current best available evidence and with good faith” ([38], p.7)	5	[26, 29, 32, 38, 45]
Reciprocity	“Support individuals who undertake front-line patient care and are exposed directly to the risk of infection due to activities inherent to their role” ([26], p.7) “Reciprocity requires that society supports those who face a disproportionate burden in protecting the public good…” ([32], p.7)	5	[26, 29, 32, 38, 45]
Other ethical concepts	Other ethical concepts include dignity, paternalism and gratitude	7	[27, 28, 30, 32, 33, 38, 39]
*C. Procedural values*			
Flexibility	“*flexibility* … means that plans must be adaptable to changing circumstances” ([45], p.5)	17	[26, 29, 31–36, 38–42, 44, 45]
Fairness/consistency	“Fairness requires that resource allocation decisions are not made arbitrarily.” ([32], p.7) “duty to care requires *flexibility* and *consistency* in an effort to provide adequate and sustained health care” (emphasis in original, [45], p.5) “fair rationing criteria and fair processes must be transparently applied at all times” ([40], p.6)	16	[25–30, 32–34, 36–38, 40, 41, 44, 45]
Transparency	“… decision-making process must be clearly documented in the patient’s medical record” ([26], p.7) “the need to provide a transparent chain of responsibilities and tasks, with clearly defined times and methods” ([30], p.7)	15	[25–27, 30–34, 36, 38, 40–43, 45]
Objectivity	“Disproportionate care should be defined on a scientifically founded estimate of the expected outcome” ([43], p.2) “promote an informed decision based on objective criteria” *(“… favoriser une décision éclairée par des éléments objectifs”*; [39], p.7)	14	[25, 27, 29, 31–35, 37–39, 41, 43, 45]
Consultation	“Make decisions in a collegial manner to take into account different points of view …” (“*Tomar las decisiones de manera colegiada, de forma que puedan expresarse diferentes puntos de vista*”; [44], p.3) “decisions to withhold or withdraw life-sustaining treatments must always be discussed and shared among the healthcare staff, the patients and their proxies …” ([42], p.6)	13	[25–27, 29, 30, 32, 34, 35, 37–39, 42, 44]
Accountability	“those responsible for making the decisions are answerable for the decisions they did or did not take” ([32], p.9)	9	[27, 28, 32–34, 37, 38, 40, 42]
Proportionality	“The number of individuals who are negatively affected by the triage system should not exceed what would be required to accommodate the surge in demand” ([37], p.3) “measures taken should be proportional to the threat” ([38], p.7)	8	[32, 34, 36–38, 41–43]
Reasonableness	“Decisions should be based on best available evidence at the time … and should have a reasonable chance of working” ([32], p.9)	5	[25, 28, 31, 32, 38]
Contestability	“An appeals process for individualized triage decisions needs to be in place…” ([29], p.3)	3	[29, 32, 34]
Other	Other procedural values include compliance with regulations, trust, paternalism and procedural fairness	4	[32, 38, 44, 45]

In our analysis, we distinguish substantive ethical concepts (i.e. the ethical basis for allocating or denying access to ICU care) from procedural values (i.e. the ethical basis for the decision-making process).^2^ While we do not propose that substantive and procedural values are entirely independent, this categorisation has been well-recognised in the literature at least since Rawls [50], and usefully highlights distinct ethical issues that require different responses. The principle of respect for autonomy or self-determination is the ethical concept mentioned by the largest number of guidelines (n = 19), followed by maximising benefits (n = 14), justice as equity (n = 14), the duty to provide care (n = 11), and non-maleficence (n = 10). The most commonly occurring procedural values are flexibility (n = 17), fairness/consistency (n = 16), transparency (n = 15), and objectivity (n = 14). Table 3 lists the ethical concepts cited in the guidelines with illustrative quotations.

The guidelines differ in the number and ordering of ethical principles and concepts used. For example, BK Austria [28] lists widely used ethical principles and concepts including patient autonomy, harm minimisation and fairness; and MSSS Quebec [34] identifies five principles and concepts (i.e. maximising benefits, proportionality, transparency, effectiveness, sustainability, and equity). Both guidelines define the principles they use.

DOH Ireland guideline [32] is the only document that makes an explicit distinction between substantive ethical concepts and procedural values, listing seven substantive principles and concepts (minimising harm, proportionality, solidarity, fairness, duty to provide care, reciprocity, and privacy) and five procedural values (reasonableness, openness/transparency, inclusiveness, responsiveness, and accountability).

### Medical and Social Criteria in Prioritisation

In addition to ethical approaches and principles, the guidelines appeal to medical and social criteria for prioritising patients during a pandemic surge (see Table 4 for the full list of medical and social criteria). We argue that there is no hard distinction between medical and social criteria, and so we have classified them as *primarily medical* if they refer to clinical factors that describe a patient’s physiological and psychological state, and *primarily social* if they refer to non-clinical factors.

**Table 4:** Medical and social criteria for prioritisation including example statements, count or number of guidelines mentioning the criterion, and the guidelines mentioning the criterion

Criteria	Examples from guidelines	Count	Guidelines
*A. Primarily medical criteria*			
Balance clinical benefits and burdens	“probable outcome of the patient’s condition, the burden of ICU treatment for the patient and their family, patients’ comorbidities and wishes, and likelihood of response to treatment” ([25], p.13)	18	[25–31, 33–35, 37, 39–45]
Prioritise patients based on short-term prognosis (survival to ICU/hospital discharge), taking account of comorbidities^a^	“The prioritisation of patients should therefore be based on the criterion of clinical success” *(“Die Priorisierung von Patienten sollte sich deshalb am Kriterium der klinischen Er- folgsaussicht orientieren*”; [31], p.4) “taking into account the existence or absence of any serious concomitant pathologies that would point to a fatal prognosis (such as terminal illness with a prognosis of irreversibility, or irreversible coma)” ([33], p.3)	18	[25–31, 34–37, 39–45]
Prioritise patients based on long-term prognosis	“long-term functional status should patients survive” ([32], p.17) “ [patients] are expected to live no more than 12 months even with successful ICU treatment” ([38], p.11)	15	[26–34, 36–38, 40–42]
Do not prioritise COVID-19 patients over non-COVID-19 patients with equal need	“Similar ICU admission criteria should apply to all patients across all jurisdictions, and equally to patients with pandemic illness and those with other conditions” ([25], p. 13)	13, explicitly stated^b^	[25–29, 31–34, 39, 40, 43, 45]
Use illness severity scoring systems	“Estimation of the current severity of the patient’s condition using clinical and non-clinical elements, possibly aided by predictive mortality scores” (“*Estimation de la gravité actuelle de l’état du patient à l’aide des éléments cliniques et paracliniques à disposition, éventuellement aidée par des scores prédictifs de mortalité*”; [39], p.3)	12	[27–29, 31, 35, 37–39, 41–44]
Do not use illness severity scoring systems	“Illness severity scores are attractive … in so far as they appear to lend objectivity …. However, they do not predict outcomes in individual patients and should not be used on their own to guide treatment decisions or resource allocation at an individual patient level” ([26], p.5)	1	[27–29, 31, 35, 37–39, 41–44] [26]
Do not provide medically futile care or care that will not meet therapeutic goals	“Stop critical care treatment when it is no longer considered able to achieve desired overall goals (outcomes)” ([35], p.14) “Realistic goal of care: Any treatment must pursue a goal of care that is feasible under the given circumstances.” ([28], p.7)	12	[26–29, 31, 35, 36, 38–41, 43]
Use medical criteria for categorical exclusion	“only patients who require mechanical ventilation (or another specific intensive-care intervention, such as hemodynamic support with vasoactive agents or continuous renal replacement therapy) are to be admitted to the ICU” ([40], p.4)	9	[25, 29, 31, 34–36, 40, 41, 43]
Do not use medical criteria for categorical exclusion	“medical categories must not lead to a general exclusion of the necessary treatments” ([27], p.2)	6	[26, 27, 30, 32, 33, 39]
*B. Primarily social criteria*			
Use patient age as an admission criterion	“The age limit for the admission to the ICU may ultimately need to be set.” ([42], p.5)	12	[26, 28, 30, 34, 35, 38–44]
Do not use patient age as an admission criterion	“Categorical exclusion, e.g. on the basis of age should be avoided as this can imply that some groups are worth saving more than others and creates a perception of unfairness.” ([32], p.17)	3	[31–33]
Use presence of disability as an admission criterion	“Patient aged under 65, or any age with stable long-term disabilities (for example, cerebral palsy), learning disabilities or autism: do an individualised assessment of frailty.” ([35], decision tree)	9	[28, 29, 35, 36, 38, 39, 41, 43, 44]
Do not use presence of disability as an admission criterion	“proscrib [e] any other kind of discrimination in access to scarce treatment resources during a pandemic due to such motives as disability of any kind.” ([33], p.9)	1	[33]
Do not use social criteria	“it would not be professionally relevant to legitimise the use of sole criteria such as age or an externally attested quality of life, either from an ethical point of view or in terms of fundamental rights” ([28], p.12)	7	[26–31, 41]
Use presence of caring responsibilities as a criterion	“Consider that adults with caring responsibilities be prioritised” ([26], p.7)	5	[26, 29, 32, 34, 41]
Use social value or worth as a criterion	“Take into account the social value of the ill person” (“*Tener en cuenta el valor social de la persona enferma*”; [41], p.12)	1	[41]
Do not use social value or worth as a criterion	“the criterion of ‘social utility’ is not an acceptable criterion” (“*le critère « d’utilité sociale» n’est pas un critère acceptable*”; [39], p.8) “Priority should not be given to anyone on the basis of socioeconomic privilege, or political rank.” ([37], p.3)	6	[32, 34, 37–40]
Use lifestyle as a criterion	“ [Take into account] questions regarding social environment and lifestyle prior to illness, without forgetting that “social utility” is an unacceptable criterion” (“*questions sur l’environnement social et le mode de vie antérieur, sans oublier que le critère « d’utilité sociale» n’est pas un critère acceptable*”; [39], p.3)	1	[39]
*Other attempts at non-arbitrariness*			
Use first to arrive as a criterion	“Order in which patients come into contact with the healthcare system; namely, the date on which they were admitted to the centre in order to objectify the starting point of the patients for whom the health care system is responsible. However, this criterion must never prevail over the others…” ([33], p.3)	3	[33, 42, 43]
Do not use first to arrive as a criterion	“A triage system seems more appropriate than the ‘first come, first served’ model…” ([38], p.9)	5	[34, 36, 38, 40, 41]
Use a lottery as a criterion	“In case of comparable medical urgency, the “first come first serve” principle, and the “random” criterion, are the most useful and fair criteria” ([43], p. 3)	2	[34, 43]
Do not use a lottery as a criterion	“In the literature as lotteries, additional criteria are discussed such as «first come, first served» and prioritisation according to social usefulness. These criteria are not to be applied.” ([40], p.3)	1	[40]

^a^ The guidelines are not clear on whether they are referring to comorbidities that would be life-limiting anyway or comorbidities that make COVID less survivable

^b^ The rest of the guidelines either imply or does not mention this criterion

#### Primarily Medical Criteria

Most of the guidelines (n = 18) contain a statement referring to balancing the medical needs of the patient, the likely outcomes, the burdens of treatment and the wishes of the patient. Regarding likely outcomes, there are several criteria referring to short- and long-term prognosis or outcomes. These criteria are defined in a range of ways, including short-term survival or survival during admission (n = 18), and longer-term survival or survival post-discharge (n = 15). The presence of co-morbidities is identified as a relevant medical criterion by 15 guidelines, although it is not always clear whether co-morbidities are seen as decreasing likelihood of survival in any case (e.g. decreased survival with or without COVID-19) or decreasing likelihood of survival specifically of COVID-19 patients (e.g. co-morbidity likely to complicate COVID-19 and thus make COVID-19 survival worse).

Over half of the guidelines [27–29, 31, 35, 37–39, 41–45] use illness severity scores as a way of ranking patients. However, the ANZICSb [26] guideline claims that these types of scoring tools “do not predict outcomes in individual patients and should not be used on their own to guide treatment decisions or resource allocation at an individual patient level” ((p.5). ANZICSb’s assessment is supported by Raschke et al.’s retrospective study, which has demonstrated the poor accuracy of Sequential Organ Failure Assessment (SOFA) scores for COVID-19 patients in ICUs across several institutions in the US [51]. Some guidelines (e.g. QLD Health and RPMO France) propose using several types of scoring tools but only as decision aids, and not as the sole determinant of allocating resources.

Over half of the guidelines (13 of 21) explicitly state that patients with equal clinical need should receive equal priority, and that COVID-19 patients should not be prioritised over patients with other illnesses.

The guidelines differ markedly in their use of categorical exclusions based on medical criteria, with nine guidelines [25, 29, 31, 34–36, 40, 41, 43] recommending this approach. Examples of medical exclusion criteria include poor prospect of treatment success [31], estimated probability of mortality > 80% [34], and presence of cognitive impairment [41], among others. In contrast, six guidelines [26, 27, 30, 32, 33, 39] explicitly advise against medically based categorical exclusion. For example, BÄK Germany [27] states that “medical criteria (e.g. dementia [or] other chronic diseases) should not lead to blanket exclusion from necessary treatments”^3^ (p.2), while CNB Italy [30] states that allocation should be based on clinical criteria “without excluding anyone a priori” (p. 7).

The need to stop futile treatment and to work towards realistic goals of care is mentioned by twelve guidelines [26–29, 31, 35, 36, 38–41, 43], reflecting the focus on trying to predict prognosis and avoid giving a scarce ICU bed to a person who will not benefit from it.

Overall the medical criteria converge on treating patients with urgent medical needs who are likely to survive, where survival is measured as either short term (e.g. to discharge) or longer term (e.g. a defined number of months after discharge). The main divergences relate to how to predict prognosis (e.g. whether or not to use illness severity scoring systems) and whether or not there should be blanket exclusion criteria.

#### Primarily social criteria

The guidelines differ in their use of social criteria such as age, disability, perceived social value, or caring responsibilities, for prioritising care. Although some guidelines accept a defined range of social criteria, all except one [41] prohibit using what is described as *social value or worth* as a criterion for prioritising care. However, some guidelines recommend taking account of relevant professional responsibilities [26] such as those of healthcare professionals and others who are “essential to managing the pandemic” ([32], p.18).

Age and disability are potentially contested criteria, given that both may involve biological features as well as highly variable social and environmental factors that modify the impact of any biological features. However, there is considerable variability amongst the guidelines in their use of age and disability as criteria. Some guidelines (e.g. SEMICYUC [41] and NICE [35]) appear to appeal to a biomedical model, which has been criticised as viewing older age or disability solely as biological impairment with little consideration of the potential impact of social and environment factors [52, 53]. Most guidelines recommend using age as a criterion, but only in conjunction with other medical criteria. For example, QLD Health [38] state that use of age or “life-cycle consideration” is appropriate in limited circumstances. Four guidelines [31–34] explicitly prohibit categorical exclusion based on age, while two guidelines [35, 42] claim that age-based exclusion is ethically justified. SIAARTI [42] states that an “age limit … may ultimately need to be set”, taking a utilitarian approach for its justification (p. 5).

Only the MSCBS Spain guideline [33] explicitly *precludes* using disability as a criterion. The guideline cites constitutional rights that prohibit discrimination based on “disability of any kind” (p. 9). Three guidelines [35, 41, 44] list disability as a categorical exclusion criterion, but offer no additional justification for this recommendation. Another six guidelines [28, 29, 36, 38, 39, 43] list disability as a criterion in conjunction with other criteria, as implied in the Clinical Frailty Score (CFS).

#### Attempts at Non-Arbitrariness using Other Criteria

Several guidelines mention the use of ‘first to arrive’ or a lottery as non-arbitrary ways of allocating ICU resources when there is equal medical need or likelihood of benefit amongst patients, although they disagree on the justifiability of these approaches. Five guidelines advise against using first to arrive, with MSSS Quebec [34] citing the risk of further marginalising already disadvantaged groups. Those in support of using first to arrive [33, 42, 43] temper their position to some extent, stating that it should only be used in scenarios of “complete saturation” ([42], p. 5), or when all other criteria are equal [33, 43]. Similarly, two guidelines recommend the use of a lottery as a last resort. SAMS [40] is the only guideline that explicitly advises against using either first to arrive or a lottery as a basis for prioritising patients.

### Triage Decision-Making Process

The majority of guidelines recommend the use of a triage committee or equivalent rather than relying solely on treating ICU physicians to make admission decisions. The proposed membership and function of triage committees vary between guidelines. Just under half recommend interprofessional and interdisciplinary teams that involve ethics committees (e.g. [27, 29, 30, 33, 36, 37].), ad hoc committees or hospital administration [33, 37], nursing teams [31], and patient representatives [34, 45], among others. Several [27, 29, 34, 37, 45] outline specific steps for establishing the triage team, specifying the number, expertise and role of members; documentation and communication of triage decisions; and the process of appealing decisions. There is no consensus as to whether or not doctors caring for ICU patients should be triage team members: ANZICSa [25], ANZICSb [26] and ÖGARI [36] guidelines explicitly endorse them as part of the triage team while CCSSA [29] and QLD Health guidelines [29, 38] recommend the opposite. A large number of guidelines [30, 32, 33, 35, 40–42] offer little to no advice on triage committee membership or decision pathways.

### Points of Convergence and Divergence: A Summary

There is strong in the guidelines around an appeal to consequentialist decision making, with specific claims that ICU triage should maximise benefits for the greatest number of people by prioritising care to those most likely to benefit (e.g. DOH Ireland [32] and Ontario [37] guidelines). Our analysis makes use of a general understanding of the consequentialist approach in philosophy and bioethics, specifically the form of this that is commonly operational in healthcare contexts, which is to maximise benefits in the form of saving the largest number of lives. In their analysis, Jöbges et al. [15] and Tele Sarmento et al. [16] similarly note general agreement in appealing to the principle of maximising benefit measured by lives or life years saved.

Equally prominent is the appeal to respect patient wishes, although it is unclear as to what this entails, with only a minority of guidelines explicitly indicating that patient will—in the form of consent to be treated or advance care directive—should be incorporated into the triage process (e.g. BK Austria [28]and DIVI Germany [31] guidelines).

Most guidelines recommend equal consideration of patients regardless of their underlying pathology, with 13 guidelines explicitly advising against prioritising COVID-19 patients over other patients (i.e. all illnesses are equal). This finding on equal treatment for equal need has been identified elsewhere [15].

Another point of general agreement among the guidelines is the need to avoid discrimination by using social value or lifestyle as a criterion for categorical exclusion, and instead to focus on medical criteria when ranking patients, on the grounds that these are more objective and thus less susceptible to bias.

As described above, there are disagreements about the use and justifiability of categorical exclusions based on medical factors, and the use of age and disability as criteria. In addition the guidelines diverge on the role of ICU specialists, with some guidelines (e.g. CCSSA [29] and QLD Health[38]guidelines) recommending triage teams that are quite separate from treating ICU clinicians, while others (e.g. ÖGARI [36] guideline) recommend including clinicians involved in the care of patients being evaluated for admission.

## Discussion

This analysis of COVID-19 ICU triage guidelines raises several issues. Here we focus on three main points: the challenges to using ethical principles and concepts within guidance documents (operationalizing abstract concepts, inconsistencies); the problematic notion of objectivity used in the guidelines; and the practical and theoretical implications of guidelines that give divergent advice.

### Difficulty in Operationalising Abstract Principles

As with any practical ethical guidance, it can be challenging to operationalize abstract principles in ways that are clearly action-guiding [54]. These guidelines were developed in response to an urgent situation, and it is beyond their scope to include exhaustive explication of the ethical concepts to which they appeal. Moreover, if these guidelines aim to be action-guiding, there is a limit on the amount of backgrounding they can provide before defeating their own objective. Nonetheless, the lack of clarity about what actions should be taken in response to moral principles, such as maximising benefits or respecting the will of patients, risks undermining the utility of the guidelines, especially where these concepts are difficult to explain and translate into practice. Maximising benefits is largely understood in terms of number of lives saved, with little acknowledgement that this approach can undermine the stated commitment to treating patients with equal need equally and avoiding discrimination. Even the well-known principle of respect for autonomy is potentially problematic in this context. In contrast to the dominant operationalisation in medical ethics, it is used here mainly to refer to the patient’s right to refuse or withdraw from intensive care treatment – i.e. it is a negative right rather than a commitment to helping patients to make the best possible decision for them. Some guidelines (e.g. BÄK Germany [27], MSSS Quebec [34], and SEMICYUC [41]) frame the principle of autonomy as a motivation for regularly consulting patients’ and families’ preferences before the triage phase, as well as during hospital and ICU admission when establishing and adjusting goals of care. However, it needs to be stated explicitly that there is no suggestion that respect for patient autonomy extends to offering treatment requested by patients if they fail to meet the relevant medical and other criteria in operation.

Ethical concepts such as dignity and solidarity are difficult to explain succinctly or to translate into allocation decisions, and such difficulty raises the risk of tokenism. Dignity is briefly mentioned in several guidelines [26–28, 38, 39]. BK Austria [28] mentions the need for equal protection of “individuals and their dignity” (p.5). RPMO France [39] defines dignity as “a higher value than any other [which] must be translated into the reality of actual contexts”^4^ and “independent of [patients’] social position, or their usefulness to others”^5^ (p.7). In these examples, dignity is defined as universal, but it is unclear as to whether it is a proxy for asserting individual equality and worth – i.e. an appeal against using social criteria in allocation decisions. Similarly, solidarity appears in some guidelines. CNB Italy [30] mentions the principles of “justice, fairness and solidarity” on multiple occasions. While this guideline goes on to describe how justice and fairness should inform decision-making, it offers no further definition of solidarity and does not explain how it should inform decision-making. In other guidelines (e.g. [32, 33]) solidarity is less about ICU triage decisions than the role of members of the public in managing the pandemic. For example, MSCBS Spain [33] states that core values of solidarity, altruism and responsibility involve provision of care complemented by the “self-discipline of the entire population” (p. 9). At times, solidarity is conflated with other concepts such reciprocity. For example, the DOH Ireland guideline [32] claims that solidarity and reciprocity may justify giving priority to healthcare workers who assume risk to their own health when managing a pandemic. Given the complexities of parsing ethical concepts, guidelines that focus on procedural recommendations may have greater utility [15]. That is, we are suggesting that the inclusion of long lists of substantive ethical values may impede the usability of guidelines because it is unclear how to operationalise the values or resolve conflicts between them, leading to a lack of action guidance. As Norman Daniels have noted, emphasis on procedural values provide opportunity for those with conflicting ethical/substantive values to come to an agreement on a legitimate process that they can endorse [55].

### Internal Inconsistencies

We found internal inconsistencies in several guidelines, demonstrating the difficulty of navigating the ethical concepts grounding the allocation decisions. First, some guidelines show internal inconsistency by recommending conflicting advice based on the same criterion. For example, SAMS [40] states that allocation should be made without discrimination, that is without unjustified unequal treatment on the grounds of “age, sex, residence, nationality, religious affiliation, social or insurance status, or chronic disability” (p. 2). Despite this statement, the guideline also uses age > 85 years as a categorical exclusion (see p. 5), stating that “if one of the exclusion criteria is fulfilled,”—i.e. the criteria of age > 85 years—“the patient is not to be admitted to the ICU”. The guideline includes a footnote clarifying that “according to available data, age is a prognostic indicator”; our interpretation is that this statement is intended to justify age as an exclusion criterion. Similarly, RPMO France [39] recommends using objective criteria to avoid discrimination, while also stating that relevant criteria include social environment and previous lifestyle.^6^ In comparison, QLD Health guideline [38] offers a more nuanced recommendation for the use of age as a criterion. The guideline states that “life cycle consideration” can be added to prioritise patients < 50 years old, if all other considerations are equal. However, the guideline acknowledges that the life cycle principle can be subjective, and age alone should not be the determinant of allocation.

Second, many guidelines show internal inconsistency by utilising two or more principles that conflict with each other, without providing advice on how to resolve such conflicts. Guidelines tend to simultaneously appeal to both consequentialist and justice-based reasons, which can often pull in different directions [50]. The conflicting advice highlights the need to establish concrete steps to protect communities that are already disadvantaged, given that relevant comorbidities track socio-economic disadvantage, with the result that members of these groups enter the system with an increased likelihood of poorer prognosis [56]. For example, ANZICSb [26] proposes using “a consequentialist approach that ensures the greatest benefit and least harm for the maximum number” while also claiming that “some protections can be incorporated to ensure that vulnerable groups are not disadvantaged” (p. 5). DOH Ireland [32] states that decisions should be based on “maximising the benefit that can be gained from limited amount of resources available” and “giving due attention to fair distribution of benefits and burdens” (p. 15). Yet consequentialist approaches that maximise benefit in medicine and public health are frequently criticised as insensitive to and perpetuating pre-existing health inequities [57].

We thus agree with the growing call for the principle of justice to play a stronger role in allocation of limited resources during public health crises. In addition to avoiding bias and outright discrimination against the underserved and underprivileged, Lynette Reid calls for modifying critical care resource triage to promote justice, “even at the cost of saving fewer lives” ([58], p.528). Mello et al. are explicit in calling for the respect for and protection of disability rights during public health emergencies, discouraging use of problematic criteria that potentially discriminate against people with disability [59]. These criteria include categorical exclusions (e.g. presence or absence of disability), and consideration of quality of life and long-term life expectancy. However, we wish to emphasise that addressing inequities at the point of triage is a necessary but by no means sufficient response, as these inequities reflect systemic injustices that cannot be compensated for solely at the level of the clinic. Pandemic planning should include specific measures to ensure protection against infection for disadvantaged individuals and communities, including income support to minimise the need for risky work, adequate access to personal protective equipment for those in services deemed essential, access to healthcare in the early stages of any infection and so forth.

### Seeking Objectivity through Illness Severity Scores

Our findings highlight the tension between Avoiding both arbitrariness and overreliance on apparently objective medical criteria when allocating limited resources during the pandemic, an observation consistent with other published analysis of ICU triage guidelines, including studies by Jöbges et al. [15] and Teles Sarmento et al. [16]. Teles Sarmento et al.’s analysis acknowledges that while medical criteria are fundamental, these criteria may not be sufficient in guaranteeing equity among patients [16]. In seeking defensible non-arbitrariness and consistency, many guidelines appeal to medical criteria such as illness severity or frailty scores to provide a potentially transparent and objective way of ranking patients. Use of illness severity scores, such as the Clinical Frailty Score, has received wide support in the medical community. Wilkinson argues that using this scoring tool is ethically justifiable, as it has been validated in different countries, and is quantitatively measurable and "less subjective" than other criteria such as medical futility ([60], p. 12). However, while these allegedly objective bases of decision-making can provide a degree of consistency and transparency, the notion of objectivity that they implicitly endorse is problematic. For one thing, allegedly objective criteria such as physical frailty or cognitive state rely on human assessment which can be subjective even in measuring or interpreting the same clinical data [61]. Furthermore, pathophysiological measures are based on “normal” populations that may vary depending on the specific locations and specific groups in question, and thus contain built-in biases [62]. Mello et al. argue that categorical exclusions from access to treatment due to pre-existing functional impairments do not necessarily reflect limited survival prospects or response to treatments. Instead, such categorical exclusions demonstrate the biases that inform how the public and the medical profession judge persons with disabilities [59].

The concept of objectivity is further complicated by the difficulty of establishing a clear distinction between medical and non-medical (or social) factors. For example, RPMO France [39] encourages decision making based on “objective components” of the patient’s condition (p. 7) while including “social environment” in addition to more recognisable medical criteria such as comorbidities, frailty, nutritional state and cognitive state^7^ (p. 8). Furthermore, one reason for the ongoing debate about the justifiability of using age and disability in medical triage is due to a lack of consensus as to whether these features are medical, social, or something more complex.

Use of illness severity scoring tools such as the SOFA or the CFS can be problematic in assuming that the prognosis for any particular patient will necessarily be similar given the same criteria (such as presence of organ failure). For example, scoring tools that include only the presence or absence, and not degree of severity, of comorbidities assume that all types of comorbidity have similar presentation and similar prognosis. In addition, there is not always a reliable way of translating from degree of patient impairment or well-being to an allegedly objective score. Finally, these scoring tools have been implemented and validated for specific medical conditions, and may not be reliable in novel conditions such as COVID-19. Raschke et al., for example, report on the poorer accuracy of SOFA scoring compared to age for mortality prediction among COVID-19 patients, based on a retrospective study of patients across 18 ICUS in the southwestern US [51]. The authors conclude that an alternative approach is needed to incorporate variables specific to COVID-19 disease prognosis.

Several strategies have been proposed as potential responses to the risk of “objectivity” overpromising fairness. Newdick et al. propose replacing scoring systems with broad categorisations (from high to medium and low priority), to avoid the presumption of objectivity that goes with using quantitative scores [63]. Andrews et al. encourage establishing interdisciplinary committees, including representation from disability advocacy groups [64]. The authors argue that the pandemic has demonstrated that allegedly objective clinical criteria tend to further disadvantage people with disability, highlighting the risk of “objective” judgments simply tracking social prejudices.

Despite their drawbacks, use of these scoring systems may be justified during a public health crisis in order to ensure transparency and build public trust. Consistency is preferable to outright arbitrary decision making in order to avoid overt discrimination. During crises, scoring tools may be the best available option to make the process of allocation less arbitrary. However, this approach should be tempered by careful audit of the consequences of the scores and in particular, a detailed examination of whether their use perpetuates existing health inequities based on age, race and disability [65].

### Implications of Guidelines that give Divergent Advice

The guidelines aim to achieve consistent and ethically justifiable decision-making, but even between relatively similar countries and/or healthcare systems, the guidance varies. Such variations have practical and theoretical implications. On a practical level, variability undermines notions of a shared ethics grounding healthcare and can make decision making seem arbitrary if a patient were to receive treatment under one guideline but not another, or under one hospital or jurisdiction but not another. This challenge may be addressed by simpler and clearer guidance. For example, Jöbges et al. claim that maximising benefit and justice should be/are the only necessary two guiding principles, in which case guidelines should concentrate on how to operationalize these [15]. A move to a narrow ethical basis for triage with a focus on procedural values may lead to more useable guidelines. This claim however needs testing, not only regarding consistency of decision making but also in terms of the impact on communities, patients and professionals when care cannot be provided to all who need it, in violation of the non-pandemic ethical norms of healthcare.

On a more abstract level, the divergent advice between guidelines may be due to a complex set of factors, including the backgrounds of the authors, sociocultural context and local norms of health governance [66, 67]. Guidelines developed by bioethics committees [28, 30, 44] tend to focus on key ethical principles while providing minimal recommendations on concrete triage processes to guide allocation. Guidelines developed by medical organisations have much greater focus on procedural recommendations [31, 35, 39], identifying specific criteria at every stage of the clinical processes. One guideline developed by legal scholars [33] focuses on the legal bases of ethical concepts necessary to inform an ethical allocation process. Moreover, norms differing between disciplines are demonstrated by some guidelines using decision trees while others rely on text instructions without figure or visual guides. Medical specialty organisations tend to use decision trees or visual guides, which we assume is consistent with the perceived ease of use for medical professionals working in busy and stressed environments.

The internal inconsistencies within and disagreements between the guidelines highlight the challenge of distinguishing substantive and procedural values, thereby creating unclear guidance. There is need for greater clarity on both types of value, on the distinction between them, and on how these values are operationalised.

## Limitations

Our study has some limitations. First, by relying on a direct Google and Google Scholar search, we may have missed protocols or guidelines that have a low searchability index, although this is offset to some extent by our additional use of existing curated lists from authoritative sources. In addition, the rapidly changing context of the pandemic means that some of our included guidelines may be superseded by versions published after June 2020.

Second, the majority of the guidelines are from European countries. While efforts were made to gather protocols from other regions, specifically from low- to middle-income countries, only two such guidelines (from South Africa and the Philippines) could be retrieved during the period of search.

## Conclusion

In summary, our narrative review offers an ethical analysis of 21 guidelines for allocating scarce ICU resources during the COVID-19 pandemic. The commonest substantive ethical concepts present in the guidelines are autonomy, justice (as equality and equity), maximising benefits and minimising harm, and duty to provide care. The most frequently cited procedural values are flexibility, fairness, transparency, and objectivity. In addition to specifying the ethical basis for allocation, the guidelines rely heavily on medical criteria including illness severity scoring systems.

Our findings show that even when they share ostensibly similar circumstances, countries can vary markedly in the way they frame and express ethical guidance. Guidelines vary in their acknowledgement of the difficulty, appropriateness or justifiability of using social criteria in allocating limited resources. While there are some dissenting guidelines, majority (13 of 21) agree that frailty, evidenced by referencing the Clinical Frailty Score, is a relevant criterion in allocating ICU resources.

Our discussion focused on challenges in developing guidelines, which include difficulties in operationalising abstract principles, avoiding internal inconsistencies, and potentially problematic assumptions about objectivity. Future guidelines may avoid some of these challenges by offering simpler and clearer guidance, with appeal to fewer ethical concepts. We recommend adoption of a clear substantive position, together with guidance on ethically justifiable procedures to implement this.

Regarding the tension between maximising utility and justice, we argue that the consequences of structural inequities cannot be appropriately or comprehensively rectified at the point of ICU allocation. Unjust decision-making in ICU triage should be viewed as a Compliance withgsymptom of a more systemic problem that requires a systemic response. In particular, inequities should be addressed in clinical decision making in conjunction with necessary community responses. Pandemic planning should thus focus more on preventative interventions in which marginalised communities are prioritised. These preventative interventions may include rapid development of culturally and language appropriate public health interventions for CALD communities, improved healthcare access for socioeconomically disadvantaged groups, and dedicated support for people with disability, among others.

Empirical studies are needed to investigate the usability or impact of the current cohort of guidelines in order to provide feedback to guideline developers. Such studies should include qualitative analyses of the value of features such as decision aids, and potential variations in the application of illness severity scores.

Our ethical analysis of 21 COVID-19 ICU triage guidelines illuminates in detail the tensions and disparities that complicate the process of allocating scarce ICU resources during the pandemic. There is a delicate balance between providing a useable decision aid while also offering an ethically justifiable foundation for those decisions to patients and their families, healthcare professionals, and the wider population.

## Data Availability

All documents included for analysis are available online.

## Footnotes

1 We used the latest versions available during the search performed in May–June 2020.

2 For more information about the substantive-procedural distinction, see Normal Daniels’ discussion of fair process and legitimacy in patient selection [49].

3 “*medizinisch geprägte Kategorisierungen (z. B. Demenz, andere chronische Erkrankungen) dürfen nicht zu einem pauschalen Ausschluss von erforderlichen Behandlungen führen”* (p. 2).

4 “… *la dignité humaine (qui) est une valeur supérieure à toute autre doit être traduite dans la réalité des situations de fait*” (p. 7; footnote 6).

5 “*indépendante de sa position sociale, ou de song utilité pour les autres*” (p. 7; footnote 6).

6 *État préalable sous-jacent du patient: comorbidités, fragilité, autonomie, âge, état de nutrition, état cognitif et environnement social, évalués à l’aide des éléments suivants* (Patient’s pre-existing underlying conditions: comorbidities, frailty, autonomy, age, nutritional status, cognitive state and social environment, assessed using the following parameters. p. 8).

7 “*comorbidités, fragilité, autonomie, âge, état de nutrition, état cognitif et environnement social*” (p. 8).
